# Editorial: Elderly patients and lymphoma

**DOI:** 10.3389/fonc.2025.1568846

**Published:** 2025-03-27

**Authors:** Maria S. Infante, José-Angel Hernández-Rivas

**Affiliations:** ^1^ Hematology Department, University Hospital Infanta Leonor, Madrid, Spain; ^2^ Department of Medicine, Complutense University, Madrid, Spain

**Keywords:** lymphoma, elderly patients, management - healthcare, target therapies, geriatric assessment

Lymphoma, particularly in elderly patients, presents unique challenges due to its different subtypes, complex biology and the intersection of age-related morbidities with treatment regimens (1). Recent research featured in this Research Topic highlights significant problems in understanding prognostic factors, therapeutic strategies and novel personalized approaches for elderly patients.

Here, we distill key insights from four studies that contribute to this growing field of knowledge.

The systematic review by Zeng et al. explored the prevalence and the prognostic implications of sarcopenia in hematological malignancies, especially diffuse large B-cell lymphoma (DLBCL) and acute myeloid leukemia (AML). This meta-analysis showed a prevalence of sarcopenia of 44.5% with higher rates in older patients. Sarcopenia was found to be associated with significantly worse overall survival (HR 1.821) and progression-free survival (HR 1.703). These findings suggest that incorporating sarcopenia assessments into prognostic models could optimize therapeutic management to address frailty in older patients.

Meanwhile, Wang et al. provided a comprehensive review of emerging therapies for relapsed/refractory (RR) marginal zone lymphoma (MZL), which often affects the elderly population. They highlighted the emergence of targeted therapies such as newer anti-CD20 monoclonal antibodies (obinutuzumab), small molecule kinase inhibitors (ibrutinib and zanubrutinib), and immunomodulators (lenalidomide). These therapies not only address the limitations of conventional treatments but also demonstrate improved efficacy and better tolerability profiles. However, significant challenges remain, such as the management of the toxicities of these new drugs and understanding the mechanisms of histologic transformation and treatment resistance.

Wang et al. presented a compelling case report of anaplastic lymphoma kinase (ALK) positive large B cell lymphoma, a rare and aggressive lymphoma subtype with poor prognosis. This study demonstrated the efficacy of combining alectinib, an ALK inhibitor, in combination with brentuximab vedotin (anti-CD30 antibody) and chemotherapy (VRCD: bortezomib, lenalidomide, cyclophosphamide and dexamethasone*).* The patient achieved a complete response and underwent successful autologous hematopoietic stem cell transplantation. The tailored approach emphasizes the potential of combining targeted therapies in the treatment of a specific subset of lymphomas.

Finally, Esteghamat et al. in an extensive review highlighted advancements in the treatment of older adults with R/R DLBCL. CAR T-cell therapy shows high response rates in this population, sometimes outperforming those of younger groups, as seen in studies like PILOT. Bispecific antibodies, such as glofitamab and epcoritamab, provide effective, less toxic alternatives. Autologous HCT remains viable with improved outcomes in carefully selected elderly patients. Despite these advancements, CAR T-cell therapy remains underused in patients aged 75 years and older, and the risk of secondary malignancies after cellular therapies warrants attention.

CAR-T cell therapy has shown significant efficacy in the treatment of RR DLBCL, including in older adults (>65 years), with high response rates and durable remissions. Early detection and management of side effects such as cytokine release syndrome (CRS) and neurotoxicity (ICANS) have improved its safety profile. Moreover, bispecific antibodies (glofitamab, epcoritamab, mosunetuzumab, odronextamab and others) are emerging as effective and safer alternatives for elderly patients, with less toxicity compared to CAR T-cell therapy, making them suitable for frail patients. Consolidation with high dose chemotherapy and autologous HCT remains a viable option in elderly patients selected based on fitness and disease sensitivity, although its utility is limited by higher toxicity and relapse rates.

In conclusion, the studies included in this Research topic share important points in the management of elderly patients with lymphoma: the need to achieve a balance between efficacy and safety in these patients, who often cannot tolerate high intensity regimens. The integration of new targeted therapies and the use of a comprehensive geriatric assessment or prognostic score (e.g., ePrognosis) may enhance a personalized approach to elderly patients.

In terms of future directions, the findings of these studies highlight crucial gaps and opportunities to improve the management of elderly lymphoma patients. Key recommendations include:

- Standardized Metrics: Establish universal diagnostic and prognostic criteria, particularly for sarcopenia and molecular markers.
- Targeted Therapies: Expand the use of targeted agents with manageable safety profiles, especially in frail patients.
- Novel therapies, particularly CAR T-cell therapy, demonstrate comparable outcomes in older adults compared to younger cohorts, underscoring their potential for curative treatment in this age group
- Clinical Trials: Prioritize the inclusion of elderly patients in clinical trials to better understand age-specific outcomes.
- Multidisciplinary Approaches: Incorporate geriatric assessments, cardio-oncologists, pharmacists, and nurses into treatment planning to optimize therapeutic benefits while minimizing risks.
